# *De novo* assembly of the common marmoset transcriptome from NextGen mRNA sequences

**DOI:** 10.1186/2047-217X-3-14

**Published:** 2014-09-19

**Authors:** Mnirnal D Maudhoo, Dongren Ren, Julien S Gradnigo, Robert M Gibbs, Austin C Lubker, Etsuko N Moriyama, Jeffrey A French, Robert B Norgren

**Affiliations:** 1 Department of Genetics, Cell Biology and Anatomy, University of Nebraska Medical Center, Omaha, Nebraska 68198, USA; 2 Callitrichid Research Center, Department of Psychology, University of Nebraska, Omaha, USA; 3 Key Laboratory for Animal Biotechnology of Jiangxi Province and the Ministry of Agriculture of China, Jiangxi Agricultural University, Nanchang 330045, China; 4 School of Biological Sciences & Center for Plant Science Innovation, University of Nebraska-Lincoln, 403 Manter Hall, Lincoln, Nebraska 68588-0118, USA

**Keywords:** *Callithrix jacchus*, Common marmoset, Transcriptome, mRNA-seq, Assembly

## Abstract

**Background:**

Nonhuman primates are important for both biomedical studies and understanding human evolution. Although research in these areas has mostly focused on Old World primates, such as the rhesus macaque, the common marmoset (*Callithrix jacchus*), a New World primate, offers important advantages in comparison to other primates, such as an accelerated lifespan. To conduct Next Generation expression studies or to study primate evolution, a high quality annotation of the marmoset genome is required. The availability of marmoset transcriptome data from five tissues, including both raw sequences and assembled transcripts, will aid in the annotation of the newly released marmoset assembly.

**Findings:**

RNA was extracted from five tissues: skeletal muscle, bladder and hippocampus from a male common marmoset, and cerebral cortex and cerebellum from a female common marmoset. All five RNA samples were sequenced on the Illumina HiSeq 2000 platform. Sequences were deposited in the NCBI Sequence Read Archive. Transcripts were assembled, annotated and deposited in the NCBI Transcriptome Shotgun Assembly database.

**Conclusions:**

We have provided a high quality annotation of 51,163 transcripts with full-length coding sequence. This set represented a total of 10,833 unique genes. In addition to providing empirical support for the existence of these 10,833 genes, we also provide sequence information for 2,422 genes that were not previously identified in the Ensembl annotation of the marmoset genome.

## Data description

### Background

Nonhuman primates have, and will continue to be, important translational biomedical models for normative biological and behavioral phenomena, and for exploring atypical and/or pathological states relevant to human health and wellbeing [1]. In the past, Old World primates (e.g., rhesus macaques) and hominoid primates (e.g., chimpanzees) have been widely utilized by the biomedical community as the primary nonhuman primate models, justified primarily on the basis of the close phylogenetic relatedness of these species to human beings, and hence high homology in biological and behavioral traits. However, Old World and hominoid primates are long-lived and costly to maintain, making the use of these primates in longitudinal research problematic from both scientific and financial perspectives.

The New World marmoset monkey (Order Primates, Parvorder Platyrrhini, Family Cebidae, Subfamily Callitrichinae) constitutes an important alternative model to Old World and hominoid primates. From a management and safety perspective, marmosets are easier and less expensive to maintain and pose substantially lower potential for zoonoses than Old World and hominoid primates [2]. These small-bodied monkeys have accelerated life histories relative to other primates, reaching reproductive age at approximately 12–18 months of age, and an average captive lifespan of seven years, thus making developmental and longitudinal studies more practicable [3]. The marmoset has proven utility as a model in a wide range of biomedical research, including immunology, reproduction, neuroscience, and developmental biology (reviewed in [2-4]). Furthermore, genomic research with this species has already yielded important insights [5,6].

A draft version of the marmoset (*Callithrix jacchus*) assembly has recently been published [6]. To conduct Next Generation gene expression studies and to study primate evolution, a high quality annotation of the marmoset genome is required. Here, we present marmoset transcriptome data from five tissues, including 30.2 Gb of raw sequences and 209,966 assembled transcripts representing the full length CDS of 10,833 unique genes. These resources should be helpful in the annotation of the newly-released marmoset assembly.

### Samples

Two 2-year-old common marmosets (*Callithrix jacchus*), (animal number 2218, female and animal number 2219, male) were euthanized due to gastrointestinal illness and associated weight loss. Marmosets were humanely euthanized with appropriate veterinary supervision according to procedures approved by the UNO/UNMC Institutional Animal Care and Use Committee (#12-099-12). The animals were initially anesthetized with gaseous isoflurane, and then received a lethal overdose of sodium pentobarbital (i.v., 150 mg/kg). Immediately upon deep sedation, tissues were collected, placed in cryovials and immediately frozen in liquid nitrogen. The cryovials were stored at -80°C. Tissues included: skeletal muscle, bladder and cerebral hippocampus (from animal number 2218) and cerebellum and cerebral cortex (from animal number 2219).

### RNA extraction

Frozen tissues were thawed and total RNA was extracted with TRIzol and purified with an RNeasy Minikit (Qiagen). Extracted RNA was stored at -80°C until analysis. RNA extraction quality was assessed with an Agilent 2100 Bioanalyzer using the RNA 6000 Nano Kit (Agilent). All samples used had RNA Integrity Numbers greater than 8.0.

### Sequencing

All five RNA samples were sequenced on the Illumina HiSeq 2000 platform (paired-end, 101 bp reads). A total of 30.2 billion bases of sequences were deposited in the NCBI Sequence Read Archive (SRA) database (Table 1).

**Table 1:** Sequence and assembled transcript accessions

**Tissue**	**Animal ID**	**SRA accessions**	**TSA accessions**
Skeletal muscle	2218	SRX285593	GAMQ01000001-GAMQ01033528
Bladder	2218	SRX285538	GAMT01000001-GAMT01041104
Hippocampus	2218	SRX285591	GAMS01000001-GAMS01044498
Cerebral cortex	2219	SRX285592	GAMR01000001-GAMR01044112
Cerebellum	2219	SRX285594	GAMP01000001-GAMP01046724

SRA, Sequence Read Archive; TSA, Transcriptome Shotgun Assembly.

### Transcriptome assembly

Reads were filtered to remove genomic contamination. To accomplish this, the reads were aligned with human RefSeq (version 57) mRNA sequences using BLAST (BLAST + v2.2.25) [7]. All reads with a reported alignment length of less than 87 and greater than 115 nucleotides were removed from the input file. We also removed a read if its mate was removed.

Filtered reads were assembled into transcripts from Velvet (v1.2.09) [8] and Oases (0.2.08) [9] (Table 2). The k-mer value used in velveth was 31; the coverage cutoff and expected coverage was set to “auto” in velvetg. The assembled contigs from velvetg were then passed to Oases, a transcriptome assembler [9]. Default parameters were used for Oases.

**Table 2:** Submitted contig statistics

**Tissue**	**No. contigs**	**N50**	**Mean length**
Skeletal muscle	33,528	2,441	1,429
Bladder	41,104	2,662	1,522
Hippocampus	44,498	2,819	1,603
Cerebral Cortex	44,112	2,926	1,615
Cerebellum	46,724	2,935	1,578

This strategy was designed to avoid false positives. However, because we used human transcripts to identify orthologs, we will likely miss annotating potential marmoset-specific genes.

### Annotation

We used our assembled marmoset transcripts as queries with BLASTx [10] (BLAST + v2.2.25) to identify orthologous human RefSeq proteins. We used the full-length coding sequence (CDS) ranges obtained from the BLASTx output files to derive putative marmoset protein sequences. Only transcripts with both start and stop codons were annotated. In addition, only transcripts with conceptually derived proteins with alignment gap lengths of less than or equal to 15, protein length differences of less than or equal to 15, and protein identities of greater than or equal to 70% with respect to their human orthologs were annotated. Both annotated and unannotated transcripts were deposited in the TSA database (Table 1).

A total of 209,966 transcripts were submitted to NCBI. From this group, we annotated 51,163 transcripts with full-length CDS. This set represented a total of 10,833 unique genes. Conceptual translations (derived from BLASTx) had an average identity and similarity of 96.56% and 97.92% to their human orthologs, respectively.

### Comparison with Ensembl annotations

**Figure 1:**
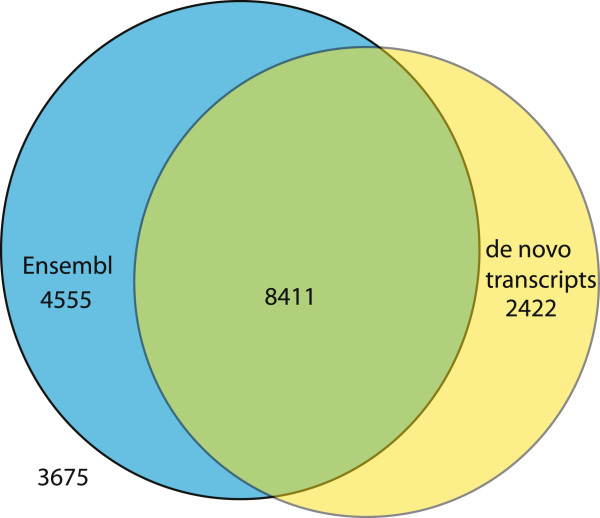
**Venn diagram illustrating the relationship between Ensembl and *de novo *transcript annotations of the marmoset genome.** There are a total of 19,063 unique human gene symbols for protein coding genes. 8,411 unique gene symbols were found in both Ensembl and our *de novo* transcripts for the marmoset. 4,555 gene symbols are found only in the Ensembl annotation and 2,422 gene symbols were found only in our de novo transcripts. 3,675 human gene symbols were not found in either Ensembl annotations or our *de novo* transcripts for the marmoset.

There were a total of 43,771 proteins in the Ensembl marmoset database (Ensembl genes 76, C_jacchus 3.2.1). These represent 15,638 unique genes. 9,901 transcripts did not contain a start codon and 7,778 did not contain a stop codon. A total of 29,364 transcripts contained both start and stop codons. 7,642 proteins were assigned the gene symbol “unk” indicating that the gene identity was unknown. A total of 25,788 transcripts had start and stop codons and a known gene identity. These transcripts represented a total of 12,966 unique genes. We used gene symbols to identify which genes were represented in both the Ensembl annotations and in our *de novo* transcripts, as well as genes that were private to each annotation (Figure 1). We found that our *de novo* transcripts add 2,422 genes that were not previously annotated by Ensembl as complete proteins. We should point out that our set of genes represents transcripts for which we could create full-length coding sequence with the Velvet-Oases pipeline. This figure does not represent all of the transcripts expressed in our datasets.

## Availability of supporting data

The datasets supporting this article are available at the National Center for Biotechnology Information (NCBI) accessioned under Bioproject ID: 203643. Illumina sequences were deposited in the SRA under accessions [SRX285593, SRX285538, SRX285591, SRX285592, SRX285594]. Assembled transcripts were deposited in the Transcriptome Shotgun Assembly (TSA) database under accessions [GAMQ01000001-GAMQ01033528, AMT01000001-GAMT01041104, GAMS01000001-GAMS01044498, GAMR01000001-GAMR01044112, GAMP01000001-GAMP01046724]. Supporting BLASTx results, and ISA-TAB metadata are also available from the *GigaScience* database [11].

## Abbreviations

BLAST: Basic local alignment search tool; CDS: Coding sequence; RefSeq: Reference sequences; SRA: Sequence read archive; TSA: Transcriptome shotgun assembly.

## Competing interests

The authors declare that they have no competing interests.

## Authors’ contributions

MDM performed the transcript assemblies, bioinformatics analyses and contributed to the writing of the paper; DR performed the RNA extractions; JSG, RMG and ACL performed bioinformatics analyses; ENM supervised bioinformatics analyses and contributed to the writing of the paper; JAF contributed to the writing of the paper; RBN conceived the study, supervised transcriptome assembly, annotation and other bioinformatics analyses and contributed to the writing of the paper. All authors read and approved the final manuscript.
